# Classification of Point Cloud Data in Road Scenes Based on PointNet++

**DOI:** 10.3390/s26010153

**Published:** 2025-12-25

**Authors:** Jingfeng Xue, Bin Zhao, Chunhong Zhao, Yueru Li, Yihao Cao

**Affiliations:** 1 Qingdao Huanghai University, Qingdao 266555, China; 2 School of Geology and Geomatics, Tianjin Chengjian University, Tianjin 300384, China; 3 Lerner College of Business and Economics, University of Delaware, Newark, DE 19716, USA; 4 Key Laboratory of Geographical Process and Ecological Security of Changbai Mountain, Ministry of Education, School of Geographical Sciences, Northeast Normal University, Changchun 130024, China

**Keywords:** point cloud data, point cloud data classification, deep learning, PointNet++, Sydney urban objects dataset

## Abstract

**Highlights:**

**What are the main findings?**

- Effective Dataset & Augmentation Strategies: Farthest Point Sampling preserves features better than random sampling, rigid transformations enhance diversity, and noise injection improves authenticity. Point filling significantly outperforms zero-padding (86.49% train/98.23% max test acc vs. 66.86%/79.89%).
- Optimized Model Performance: Using optimal hyperparameters (lr = 0.00075, batch = 6, Adam, PointNet++ MSG), the model achieved 86.26% avg train acc (98.54% max) and 97.41% test acc. Most categories (e.g., biker, excavator) performed excellently, while a few (e.g., building, traffic lights) had low recall due to sample issues; minor misclassifications from small dataset/imbalance were mitigated by hyperparameter tuning.

**What are the implications of the main findings?**

- This study presents a viable high-precision classification technique for autonomous driving and map creation, providing a reliable reference for related research and applications in complex road environments.
- The findings provide offers a reusable framework for road scene point cloud dataset construction, data augmentation, and PointNet++ modification, supporting similar 3D data processing tasks.

**Abstract:**

Point cloud data, with its rich information and high-precision geometric details, holds significant value for urban road infrastructure surveying and management. To overcome the limitations of manual classification, this study employs deep learning techniques for automated point cloud feature extraction and classification, achieving high-precision object recognition in road scenes. By integrating the Princeton ModelNet40, ShapeNet, and Sydney Urban Objects datasets, we extracted 3D spatial coordinates from the Sydney Urban Objects Dataset and organized labeled point cloud files to build a comprehensive dataset reflecting real-world road scenarios. To address noise and occlusion-induced data gaps, three augmentation strategies were implemented: (1) Farthest Point Sampling (FPS): Preserves critical features while mitigating overfitting. (2) Random Z-axis rotation, translation, and scaling: Enhances model generalization. (3) Gaussian noise injection: Improves training sample realism. The PointNet++ framework was enhanced by integrating a point-filling method into the preprocessing module. Model training and prediction were conducted using its Multi-Scale Grouping (MSG) and Single-Scale Grouping (SSG) schemes. The model achieved an average training accuracy of 86.26% (peak single-instance accuracy: 98.54%; best category accuracy: 93.15%) and a test set accuracy of 97.41% (category accuracy: 84.50%). This study demonstrates successful road scene point cloud classification, providing valuable insights for point cloud data processing and related research.

## 1. Introduction

Point cloud data, as an important carrier of three-dimensional geographic information, plays a significant role in computer vision and is widely used in various practical applications [1]. The classification of road point cloud data aims to identify and distinguish different objects in road scenes, enabling tasks such as traffic condition analysis, vehicle recognition, and obstacle detection [2,3].Consequently, this technology is of great importance in areas such as autonomous driving systems and high-precision map creation [4,5]. With the continuous advancement of urbanization, the volume of road point cloud data acquired is also increasing rapidly. How to efficiently process this massive amount of data and perform accurate analysis has become a core issue that needs to be addressed in the construction of digital cities.

Previous methods for road point cloud classification typically relied on the extraction of image visual features and the setting of fixed thresholds [6]. However, in complex and dynamic road environments, these methods often fail to achieve the desired results. For instance, road point cloud data collected at night [7] or under adverse weather conditions [8] may have limited resolution, leading to difficulties in the classification process using traditional methods. Many traditional algorithms lack adaptability to such environmental changes, significantly reducing the accuracy of the classification results. Therefore, how to handle the characteristics of data in complex environments and improve classification accuracy has become a major challenge in current road point cloud data analysis [9,10]. With the growing popularity of deep learning, point cloud classification methods based on neural networks have also emerged. Zhang proposed a point geometry transformation method for three-dimensional point cloud classification and partial segmentation. By exploring underlying geometric structures, their method effectively handles incomplete and occluded data [11]; Inspired by biology, Hu developed a complementary dual-awareness network modeled after the neural system of the human visual system, aimed at enhancing the ability to perceive 3D objects in real scenes. This network utilizes a carefully designed variational resolution and field of view, as well as multiple basic perception units stacked in a complementary hierarchical system to achieve a complementary dual-awareness mechanism; Zhou addressed the issues of insufficient weighted feature information in attention mechanisms and the discrepancy between weighted results and task expectations by introducing a feature feedback repair module to mitigate information loss during feature embedding. Additionally, an efficient hierarchical local feature encoder was used to extract and aggregate local features from point clouds of different scales, significantly enhancing the model’s ability to represent geometric structures [12]. The aforementioned methods have made varying degrees of progress in point cloud data processing. However, existing research focuses more on the design and optimization of the models themselves. Although they are advanced in global or local information extraction, few studies consider the actual effectiveness of the models in practical applications, especially in fully exploiting the deep semantic information within road scene point cloud data.

PointNet [13] is a mature neural network for point cloud data processing that directly processes point cloud data, making full use of the rich semantic information contained within the three-dimensional data. The design of PointNet revolves around three key aspects: Firstly, to address the disorderly nature of point cloud data, PointNet combines a maximum pooling layer with multiple layers of perceptrons (MLPs) to create a symmetric network structure, ensuring that the order of input data does not affect the processing outcome. Secondly, PointNet introduces a combination of global features and individual point cloud features for data processing tasks, effectively improving the efficiency of feature extraction. Lastly, to minimize the impact of geometric transformations (such as translation and rotation) on the processing results, PointNet incorporates a small network called T-net to perform affine coordinate transformations. A specific loss function is used to make the affine transformation matrix approach an orthogonal matrix, ensuring the accuracy and stability of the transformations. However, PointNet has limitations in feature extraction, as it does not fully consider the semantic features of neighboring points, which are crucial in spatial data analysis. To overcome this deficiency, PointNet++ [14] builds upon PointNet by adopting a hierarchical network structure. The core idea of this method is to iteratively apply PointNet to local areas to extract local features from the point cloud data, thereby constructing a multi-layered neural network architecture that enables multi-level feature extraction while maintaining invariance to translations. After this process, multi-scale aggregation (MSG) and multi-resolution aggregation (MRG) are used to enhance the model’s ability to handle point cloud data with non-uniform density, making the model more robust and significantly improving the performance of point cloud data processing. Compared with graph-based methods such as DGCNN, which rely on the explicit construction of dynamic neighborhood graphs to define local edge relationships, and convolution-based approaches such as KPConv, which depend on predefined kernel point configurations in Euclidean space, PointNet++ adopts a fully end-to-end hierarchical feature learning framework that directly operates on unordered point sets. In DGCNN and KPConv, local geometric relationships are modeled through intermediate structural representations, such as dynamically updated graphs or manually parameterized convolutional kernels, which introduce additional modeling assumptions beyond the raw point coordinates. In contrast, PointNet++ integrates sampling, grouping, and feature aggregation within a unified end-to-end pipeline, enabling stable local feature extraction under sparse or complex point cloud conditions without requiring explicit graph topology construction or kernel geometry specification. These characteristics make PointNet++ a robust backbone for point cloud classification under heterogeneous sampling densities.

Point cloud data classification based on deep learning has made significant progress in various fields, but existing models still face considerable challenges when processing road scene point clouds. The complexity and variability of road environments require classification algorithms to accurately identify different types of objects in road scenes, such as vehicles, vegetation, and traffic signs [2,15]. However, current algorithms still fall short when dealing with complex scenarios, especially when dealing with cases where objects are closely adjacent. Accurately distinguishing the categories of objects in road scene point clouds remains a critical issue that needs to be addressed in the field of road point cloud classification. From this perspective, the present study leverages a proven point cloud learning architecture as a foundation to investigate practical strategies for handling heterogeneous, sparse, and structurally complex point cloud data, with a particular emphasis on multi-source dataset construction and systematic processing pipelines for road scene classification, providing practical insights for robust and reproducible point cloud analysis in real-world applications.

## 2. Research Data

### 2.1. ModelNet40 Dataset

The Princeton ModelNet project provides researchers in the fields of computer vision, computer graphics, robotics, and cognitive science with a comprehensive and high-quality collection of 3D CAD model objects. The project establishes a standardized vocabulary for object categories by compiling lists of common objects and combining statistical data obtained from the SUN database. The team queried relevant terms for each object category using an online search engine and collected 3D CAD models for each category, and converted them into point cloud data. The ModelNet40 dataset includes 40 categories and a total of 12,311 3D CAD models, which are used to train 3D deep learning projects. The dataset is divided into a training set and a test set, with the training set containing 9843 model files and the test set containing 2468 model files. Notably, a key feature of the ModelNet40 dataset is that all the point cloud data is generated by scanning 3D CAD models, ensuring that the number of points in each point cloud file is uniformly contains 10,000 points. This characteristic guarantees the uniformity and standardization of the dataset when training deep learning models. The organization of the point cloud data in the ModelNet40 dataset is detailed in Table 1.

The Sydney City Objects Dataset (originally named Objects4, Sydney Urban Objects Dataset-ACFR-The University of Sydney) was released in 2013 by Alastair Quadros, James Underwood, and Bertrand Douillard. This dataset contains various common urban road objects scanned with a Velodyne HDL-64E LiDAR (manufactured by Velodyne LiDAR, San Jose, CA, USA) in the central business district of Sydney, Australia. It includes 631 objects, covering multiple categories such as vehicles, pedestrians, traffic signs, and trees. The dataset is primarily used for testing matching and classification algorithms, aiming to simulate non-ideal sensing conditions in real urban environments, particularly occlusion. Each object in the dataset is provided in three storage formats, with the simplest being ASCII CSV format (objects/. csv), while the more compact and faster-processing format is binary CSV (objects/. bin). The organization of point cloud data in the Sydney Urban Objects Dataset is shown in Table 2.

### 2.2. ShapeNet Dataset

ShapeNet (ShapeNet) is a richly annotated, large-scale 3D shape dataset comprising multiple subsets. ShapeNetCore is a curated subset containing approximately 51,300 unique 3D models spanning 55 common object categories, with each model manually verified for category labels and alignment information. It comprehensively covers all 12 object categories from the PASCAL 3D+benchmark dataset and is widely used in 3D computer vision tasks. Another subset, ShapeNetSem, includes 12,000 models distributed across 270 categories, which provides more detailed annotations. Beyond category labels and alignment information, it additionally contains physical dimensions, estimated material composition by category, as well as overall volume and weight data. These subsets offer comprehensive 3D data support for diverse research needs.

### 2.3. Dataset Integration

This study realizes the classification of road scene feature point cloud data based on the PointNet++ architecture. Among the aforementioned datasets, the ModelNet40 dataset organizes data by category and supports data querying via indexing mechanisms. This data organization mode is highly consistent with the point cloud classification goals of this study. Therefore, this study determines to adopt the data organization format of ModelNet40. Nevertheless, the categories covered by the ModelNet40 dataset are not fully applicable to road scene classification tasks. For example, categories such as airplanes and cups are irrelevant to the training process of road scene point cloud classification, and the feature categories required for this study are relatively insufficient in this dataset.

To address this issue, this study further selected the Sydney Urban Objects Dataset, which reflects the actual road environment through LiDAR scan data from actual road scenes. To enable PointNet++ to properly read and process this data, the study performed data extraction, reorganization, and index creation on the Sydney Urban Objects Dataset. Additionally, given the relatively small sample size of the Sydney Urban Objects Dataset and the insufficient number of samples in certain feature categories, this study also extracted suitable training samples from the ModelNet40 and ShapeNet datasets. After data processing, these samples were incorporated into the road scene feature classification dataset (Table 3).

## 3. Methodology

### 3.1. Building a Road Scene Classification Dataset

Although the ModelNet40 dataset has a well-organized structure, there are certain differences in the recorded information compared to other point cloud datasets. Specifically, ModelNet40 does not include common point cloud data attributes such as timestamps and laser intensity, and the PointNet++ architecture is not designed with specialized convolutional layers to process these additional types of information. Moreover, the point cloud data in the Sydney Urban Objects Dataset is stored in both ASCII CSV and binary CSV formats, which differ significantly from the txt format used in the ModelNet40 dataset. As a result, the PointNet++ architecture cannot directly process the point cloud data from the Sydney Urban Objects Dataset. To address this, the spatial coordinates of the point clouds must first be extracted from the original dataset and converted into txt format. This process is accomplished using a Python-based batch data processing script (Python 3.7.12, PyTorch 1.12.0, CUDA 11.6). During this procedure, samples of the same category from multiple point cloud datasets are numbered, reclassified, and assigned appropriate delimiters and labels. This results in the creation of a road point cloud dataset that meets the processing requirements of PointNet++ and follows a data organization style similar to the ModelNet40 format, namely the Road Scene Classification Dataset.

### 3.2. Data Augmentation and Modification

**Figure 1:**
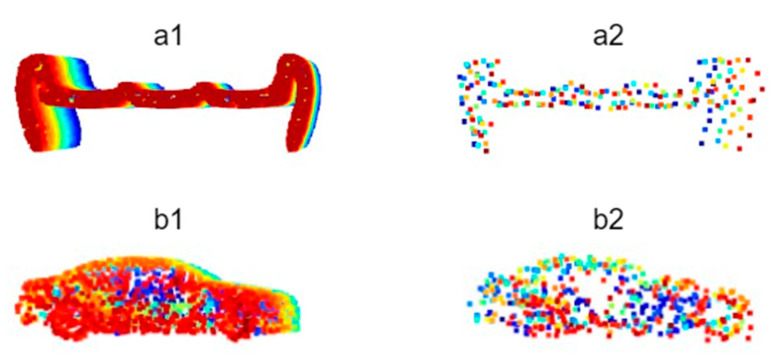
Comparison of point cloud data before and after processing with FPS and Gaussian noise addition ((**a1**): Original bench point cloud, (**a2**): Processed bench point cloud; (**b1**): Original car point cloud, (**b2**): Processed car point cloud).

Deep learning requires a large amount of sample data. However, measured sample data are influenced by various measurement conditions, such as noise caused by the inherent precision limitations of laser scanners [16], gaps in the point cloud data of target objects due to occlusion by foreground features [17], and the difficulty for fixed scanners to capture the diverse spatial characteristics of the same object from different angles [18]. Under the combined influence of these factors, the samples in the dataset may not fully meet training requirements. To ensure the dataset can effectively reflect the prominent features of actual objects, this study employs point cloud data augmentation methods to expand the dataset and mitigate the impact of noise. The following measures are primarily adopted for data supplementation and augmentation:

1. Perform farthest point sampling on the augmented data and add noise:

For categories with insufficient samples or inadequate feature representation in the Sydney Urban Objects Dataset, additional data of corresponding categories were extracted from datasets such as ShapeNet and incorporated into the custom dataset to supplement the data volume. However, directly integrating point cloud data from external datasets into the road scene object classification dataset would cause significant performance degradation, as the point density in real-world road scene point clouds is significantly lower than that in model datasets like ShapeNet, and the point cloud quality is inferior due to environmental interference. This discrepancy could cause PointNet++ to disproportionately focus disproportionately on high-quality, dense samples, leading to local optima and overfitting. Consequently, this imbalance affects the network weights and reduces prediction and recognition accuracy. Studies have demonstrated that Farthest Point Sampling (FPS) is more effective than random sampling in preserving the shape features of original point clouds. The FPS algorithm selects the point farthest from the already chosen points as the next sampling point, thereby retaining the global shape information of the point cloud while minimizing the number of sampling points. In contrast, random sampling does not account for the spatial distribution of points and may omit critical shape feature points, leading to greater feature loss [19]. This study addresses this issue by applying FPS to downsample the additional data samples selected from the external datasets ModelNet40 and ShapeNet and adds random Gaussian noise with a mean of 0 and a variance of 0.1. This approach effectively reduces the risk of neural network overfitting (Figure 1).

**Figure 2:**
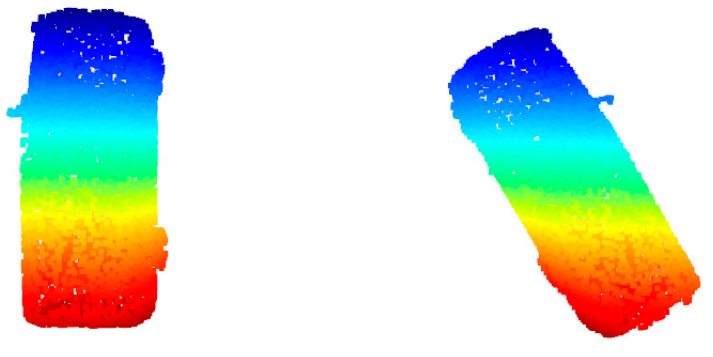
Comparison of point cloud data before and after rotating only around the Z-axis (the random rotation value for this frame of point cloud is 0.5719).

2. Applying random rigid transformations to the data:

Objects in road scenes follow certain distribution patterns, yet their poses often vary. On roads with different orientations, the movement directions of vehicles and pedestrians differ, and the alignment of pole-like features such as streetlights and buildings on either side of the road also varies. Additionally, the placement of laser scanners affects the morphology of the captured point clouds, and differences in pose can lead to variations in spatial features to some extent. Therefore, rigid transformations are essential in point cloud neural network training [20]. This study aims to feed multi-angle training samples into PointNet++. To achieve this, point cloud data of the same object category should not maintain a fixed pose when input into PointNet++. Instead, they are rotated arbitrarily around the Z-axis (i.e., the vertical axis of the road coordinate system; rotations around the X- or Y-axis are intentionally avoided, as they would generate physically implausible object poses such as overturned or vertical vehicles that cannot be observed by on-road sensing platforms). Such rotations around the Z-axis help the model better understand object shapes and structures from different perspectives, thereby increasing the diversity of training data and improving model accuracy (Figure 2).

**Figure 3:**
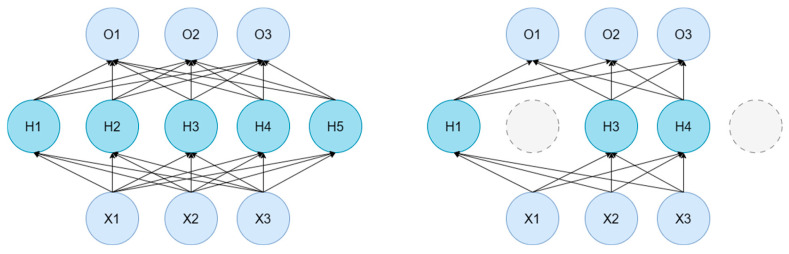
Dropout schematic diagram.

3. The processing of data within PointNet++:

In addition to dataset augmentation, PointNet++ also applies certain transformations to point cloud data during the reading process. For example, it may generate random sequences to shuffle the order of points before reading, add random noise to introduce slight perturbations to the point cloud data, apply random increments to the coordinates of each point for random translation, or multiply the entire point cloud by a scaling factor to achieve resizing. One notable data augmentation method used is called Random Dropout (Figure 3). In PointNet++, for the output of the Multi-Layer Perceptron (MLP) at each point, a subset of the output values is randomly set to zero with a certain probability. This process is referred to as dropout. Dropout ensures that only a portion of the neurons are activated during training (e.g., H1, H3, and H4 in Figure 3), reducing the co-adaptation between neurons. Thereby, it introduces randomness into the training process and mitigates the model’s propensity for overfitting on the training data.

### 3.3. PointNet++ Architecture Optimization

In Section 2.1 of this paper, the characteristics of the ModelNet40 dataset are described in detail, with particular emphasis on the fact that all point cloud files in the ModelNet40 dataset consistently consist of 10,000 points. The classification functionality of the PointNet series was developed based on this dataset, which limits its generalizability when applied to other datasets. In practical road scene point cloud data, significant occlusion and environmental interference such as weather and temperature often result in point cloud data for specific objects that rarely achieve the quality and density levels of ModelNet40 samples. Consequently, the number of points in such point cloud files often falls short of 10,000 sampled points. This inconsistency in point counts leads to alterations in the shape of point clouds when they are input into the neural network, subsequently causing dimensional errors in the tensor composed of all point clouds.

This study modifies the data reading module of PointNet++ by incorporating a point filling strategy. For road scene object targets with sparse laser footprints, the point cloud density is reasonably increased based on their geometric shape characteristics, ensuring that all point clouds input into the neural network maintain a consistent shape. Two approaches were compared: one method simply pads the data with zero vectors, while the other employs the strategy proposed in this section to modify the data preprocessing module. Specifically, zero vector padding increases the input size by inserting artificial points to satisfy the fixed input size requirement. However, since these inserted points do not correspond to actual LiDAR measurements and are located at fixed coordinate positions, we consider that this strategy may potentially introduce non physical geometric artifacts and alter local neighborhood statistics during point grouping and feature aggregation, particularly in sparse point cloud scenarios. In contrast, the point filling strategy adopted in this study satisfies the fixed input size requirement by duplicating existing points from the original point cloud without introducing new spatial coordinates. This operation preserves the spatial extent, relative point distribution, and global geometric envelope of the original object, while only adjusting the effective sampling frequency of observed points. From an architectural perspective, the potential impact of point duplication on feature learning is mitigated by the design of PointNet++, which applies shared multilayer perceptrons followed by symmetric aggregation functions such as max pooling. Under this formulation, duplicated points do not introduce new geometric extrema within local neighborhoods and therefore have a limited influence on the aggregated feature representations. Furthermore, the hierarchical structure of PointNet++, combining farthest point sampling with local neighborhood grouping, reduces the likelihood that duplicated points dominate representative point selection or distort local geometric structures. Consequently, the point filling strategy can be regarded as a geometrically conservative and structurally consistent solution for handling sparse road scene point clouds under fixed input size constraints.

Both methods were trained under identical hyperparameter conditions, and their final achieved accuracy was compared after training. Experimental results demonstrated that, during training, the simple zero-padding method achieved an accuracy of 66.8561% on the training set, with a best accuracy of 79.8872% on the test set and a best class accuracy of 47.0780%. In contrast, the point filling method achieved an accuracy of 86.4899% on the training set, with a best accuracy of 98.2323% on the test set and a best class accuracy of 89.7889%. In prediction tasks, the simple zero-padding method achieved a class accuracy of 70.3283%, while the point filling method achieved a class accuracy of 79.1751%. The experiments confirm that point filling can effectively meet the requirements of the PointNet series for reading variable-length point cloud datasets with high accuracy.

### 3.4. Hyperparameters and Optimizers

#### 3.4.1. Hyperparameters

In deep learning tasks, the values set for hyperparameters directly influence the final training outcomes, and different combinations of hyperparameters can significantly impact the final model weights [21]. This study primarily focuses on adjusting the following hyperparameters:

1. Learning Rate

Learning rate is a critical hyperparameter that controls the magnitude of parameter updates during each model training iteration. In the early stages of training, setting a high learning rate can help the model quickly approach an approximate solution. However, in the later stages, an excessively high learning rate may cause significant fluctuations in weights, making it difficult to achieve stable results, or it may cause the model to overshoot the optimal solution and fail to converge. Conversely, a learning rate that is too low may result in slow training progress in the early stages, requiring more training epochs to approach an approximate solution, though it can facilitate stable convergence in the later stages. Therefore, the learning rate needs to be dynamically adjusted during the early and late phases of training [22].

A parameter closely related to the dynamic adjustment of the learning rate is the decay rate. The decay rate controls how the learning rate gradually decreases according to a specific strategy during training. As training progresses, the learning rate is reduced in each epoch based on the decay rate. Common learning rate decay functions include exponential decay and cosine decay. Equation (1) represents the exponential decay function:

(1) $$y=A\times e^{-\lambda t}$$

In this formula, y represents a value that changes over time; A is the initial value, also known as the amplitude; λ determines the speed of the decay; and t or another continuous variable is used as the basis for the adjustment.

2. Batch Size

Batch Size refers to the number of samples input into the neural network during each training iteration. Due to limitations in GPU memory, it is not feasible to input all data into the network at once. Instead, the data must be processed in batches. For neural network training, leveraging hardware acceleration (such as the parallel computing capabilities of GPUs) allows larger batch sizes to process more samples per iteration, significantly improving the efficiency of point cloud data processing. However, larger batch sizes also demand better hardware conditions; otherwise, memory insufficiency issues may arise. Conversely, loading data in smaller batches inevitably prolongs the time required for data processing, thereby slowing down training efficiency. Nevertheless, smaller batch sizes facilitate more frequent updates of weights in the neural network, endowing the model with better generalization capabilities [21]. Therefore, when selecting the batch size, a trade-off between efficiency and performance must be made to find an optimal balance between the two.

#### 3.4.2. Optimizers

In the training process of deep learning models, the choice of optimizer significantly impacts the convergence speed and final accuracy of the model. This study considers two optimizer options: SGD (Stochastic Gradient Descent) and Adam (Adaptive Moment Estimation).

SGD is a classic optimization algorithm that calculates the gradient and updates the model parameters using one sample at a time. Its parameter update rule is as follows:

(2) $$\theta_{t+1}=\theta_{t}-\eta \nabla_{\theta }L\left(\theta_{t}\right)$$

Here, $\theta_{t}$ represents the current parameter value, η is the learning rate, and $\nabla_{\theta }L\left(\theta_{t}\right)$ is the gradient of the loss function $L\left(\theta_{t}\right)$ with respect to the parameter θ. Since SGD calculates gradients using only a single sample at a time, it offers high computational efficiency on large-scale datasets. However, SGD is prone to gradient oscillations, particularly in the early stages of training, which may lead to unstable parameter updates. To mitigate this issue, the Momentum strategy is commonly adopted. By accumulating information from past gradients, Momentum smooths parameter updates, thereby accelerating convergence and avoiding oscillations.

In contrast, Adam [23] is an adaptive learning rate optimization algorithm that combines the concepts of Momentum and RMSprop. Adam adaptively adjusts the learning rate for each parameter by calculating the first-order moment (mean) and second-order moment (variance) of the gradients. Its update rules are as follows:

Calculate the first-order and second-order moments of the gradients:

(3) $$m_{t}=\beta_{1}m_{t-1}+\left(1-\beta_{1}\right)\nabla_{\theta }L\left(\theta_{t}\right)$$

(4) $$v_{t}=\beta_{2}v_{t-1}+\left(1-\beta_{2}\right){\left(\nabla_{\theta }L\left(\theta_{t}\right)\right)}^{2}$$

Calculate the bias-corrected values for and:

(5) $${\hat{m}}_{t}=\frac{m_{t}}{1-\beta_{1}^{t}},{\hat{v}}_{t}=\frac{v_{t}}{1-\beta_{2}^{t}}$$

Update the parameters:

(6) $$\theta_{t+1}=\theta_{t}-\eta \frac{{\hat{m}}_{t}}{\sqrt{{\hat{v}}_{t}}+\epsilon }$$

Among these, β1 and β2 are the decay rates for the first-order and second-order moments, typically set to β1=0.9 and β2=0.999. ϵ is a small constant used to prevent division by zero errors. The advantage of Adam lies in its ability to adjust the learning rate for each parameter based on the mean and variance of the gradients. This makes the step sizes of parameter updates more flexible, thereby enhancing training stability, particularly in tasks with sparse gradients or significant gradient noise.

Both SGD and Adam have their respective advantages and disadvantages. SGD is simple and computationally efficient, making it suitable for large-scale datasets. However, when dealing with sparse gradients or complex loss functions, it may lead to slow convergence or oscillations. Adam effectively addresses these issues by combining adaptive learning rates with momentum, demonstrating greater stability, particularly in deep neural networks and complex tasks. Nevertheless, Adam requires careful selection of hyperparameters and may lead to overfitting on small datasets. This study will select the appropriate optimizer based on experimental test results.

### 3.5. Model Prediction Accuracy Evaluation Metrics


(7) $$Precision=\frac{TP}{TP+FP}$$

Among these, TP (True Positive) represents the number of samples correctly predicted as positive, while FP (False Positive) refers to the number of negative samples incorrectly predicted as positive. Precision is primarily used to measure the accuracy of the model, especially in tasks where misclassification of negative samples (False Positives) is a critical concern. Precision serves as a key evaluation metric in such scenarios.

Recall, also known as sensitivity, represents the proportion of actual positive samples that are correctly predicted as positive by the model. It measures the model’s ability to identify positive samples. A higher recall indicates that the model can identify most of the positive samples. The formula for recall is:

(8) $$Recall=\frac{TP}{TP+FN}$$

Among these, FN (False Negative) represents the number of positive samples incorrectly predicted as negative. Recall is primarily used to measure the coverage capability of the model, especially in tasks where missing positive samples (False Negative) is highly critical. In such scenarios, recall serves as an essential evaluation metric.

The F1 score is the harmonic mean of precision and recall, serving as a comprehensive metric that balances the trade-off between precision and recall. The F1 score is calculated using the following formula:

(9) $$F1=2\times \frac{Precision\times Recall}{Precision+Recall}$$

The F1 score ranges between 0 and 1, with a higher value indicating a better balance between precision and recall. It is particularly useful in scenarios with class imbalance, as it mitigates the bias that may arise from relying solely on precision or recall. In practical applications, depending exclusively on precision or recall may fail to provide a comprehensive reflection of model performance, especially when dealing with imbalanced classes. For instance, when negative samples significantly outnumber positive samples, precision may be very high while recall remains low, or vice versa. As a composite metric of both, the F1 score helps avoid such biases to a certain extent, making it particularly suitable for tasks that require a balanced consideration of both positive and negative prediction outcomes.

Precision, recall, and the F1 score can not only be used to evaluate individual classes but can also be extended to multi-class classification tasks. Typically, they are aggregated through methods such as weighted averaging or macro-averaging to derive a global performance evaluation.

## 4. Results

### 4.1. Hyperparameter Tuning for PointNet++ Architecture

This section tests the settings of different hyperparameters to select the optimal ones. The computer and deep learning environment configuration used in this study are shown in Table 4, and all hyperparameter tuning experiments were conducted in this environment. It should be noted that the optimal hyperparameter settings are influenced by computer hardware and environmental configurations. Therefore, when training is performed in different computing environments, the optimal parameters may vary. As a result, the hyperparameter tuning results in this study serve only as a reference. It is worth noting that the computational environment considered in this study primarily reflects an offline training and experimental setting, which differs in purpose from the on-board computing platforms used in autonomous vehicles. The hardware configuration mainly influences training efficiency, feasible dataset size, and the scope of hyperparameter exploration, but does not alter the underlying model architecture or inference logic. Once trained, the inference performance of PointNet++ is largely independent of the training hardware and is instead determined by model complexity and input size. In practical deployment, autonomous vehicle platforms are typically equipped with dedicated high-performance computing units, such as NVIDIA DRIVE series systems or automotive-grade GPUs, which provide substantially higher parallel computing capability and memory bandwidth. Under such conditions, the proposed model is expected to achieve faster inference and more stable real-time performance than in the experimental environment. Therefore, while hardware constraints may limit training scale and tuning flexibility, they do not negatively affect the applicability of the trained model, and the PointNet++ framework remains well suited for point cloud classification tasks in practical autonomous driving systems.

#### 4.1.1. Learning Rate Adjustment Experiment

**Figure 4:**
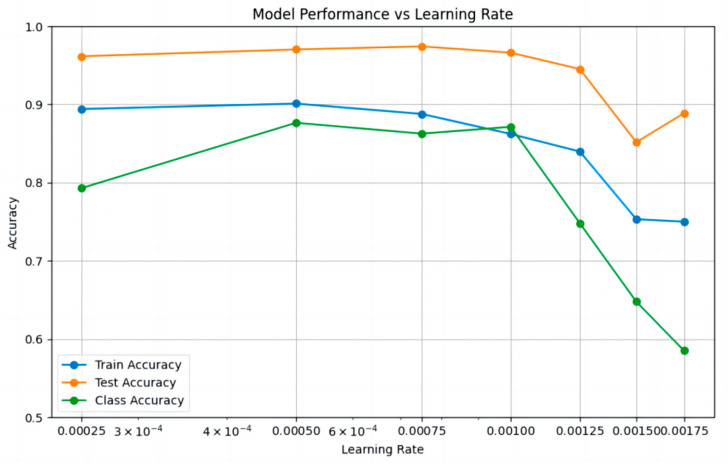
Changes in Model Training Accuracy, Test Accuracy, and Category Accuracy with Learning Rate.

In this experiment, the initial learning rate was set to 0.00025, 0.0005, 0.00075, 0.001, 0.00125, 0.0015, and 0.00175 respectively, for training. After training, the prediction results on the test set were recorded, and the number of training epochs at which overfitting occurred was statistically analyzed and plotted in Figure 4.

As shown in the figure, as the learning rate increases, the test accuracy decreases from 0.961538 to 0.888710, indicating an overall downward trend in model precision. A smaller learning rate enables more frequent weight updates, allowing the model to learn finer features. The category accuracy initially increases and then decreases with the rising learning rate, dropping sharply when the learning rate exceeds 0.001. The median values for training accuracy and test accuracy are 0.8622 and 0.9615, respectively, both achieved at a learning rate of 0.001. The median category accuracy is 0.7929, achieved at a learning rate of 0.00075. Overall, the average training accuracy is 0.8412, the average test accuracy is 0.9368, and the average category accuracy is 0.7692.

The advantages and disadvantages of each category are summarized in Table 5 to evaluate the training performance under different learning rates. The evaluation method is as follows: for each metric, if the value is above the median, one point is added; if it reaches the optimal value, two points are added. Conversely, if the value is below the median, one point is deducted, and if it is the worst case, two points are deducted. The two smallest learning rates (0.00025 and 0.0005) are penalized by one point each for being prone to local optima, while the two largest learning rates (0.0015 and 0.00175) are penalized by one point each for causing model instability.

In the experiments, the performance of different learning rates significantly influenced the model’s training accuracy, test accuracy, and category accuracy. Smaller learning rates (e. g., 0.00025 and 0.0005) generally maintained high training and test accuracy. However, due to the small learning rate, the model was prone to getting stuck in local optima, and category accuracy remained low. As the learning rate increased (e. g., 0.00075 and 0.001), both training and test accuracy gradually improved, with category accuracy performing better, particularly reaching its peak at 0.00075. However, when the learning rate further increased (e.g., 0.00125 and 0.00175), training and test accuracy began to decline, and category accuracy significantly dropped, indicating that larger learning rates may lead to difficulties in model convergence and even poorer classification performance. In summary, this study recommends setting the learning rate to 0.00075.

#### 4.1.2. Batch Size Adjustment Experiment

In this experiment, training was conducted with three different batch sizes: 4, 6, and 8. The number of epochs required to reach overfitting and the best prediction accuracy were recorded, with the results presented in Table 6. It is important to note that, due to hardware limitations, training with batch sizes larger than 8 resulted in memory overflow, causing abnormal termination of the training process. To ensure training stability, batch sizes larger than 8 were not used in this study.

The results indicate that when the batch size is 6, the model achieves the highest accuracy of 0.987719, demonstrating the best performance. While the accuracy for batch sizes 4 and 8 is also relatively high, at 0.975806 and 0.973958 respectively, it is slightly lower than that achieved with a batch size of 6.This suggests that, in this experiment, a batch size of 6 strikes a better balance between training efficiency and the model’s learning capability, leading to superior accuracy. The experiment demonstrates that as the batch size increases, accuracy generally follows a trend of first increasing and then decreasing. Conversely, an excessively small batch size results in slower training speed. Therefore, a moderate batch size of 6 is selected for training.

#### 4.1.3. Comparative Experiments on Other Parameters and Model Selection

**Figure 5:**
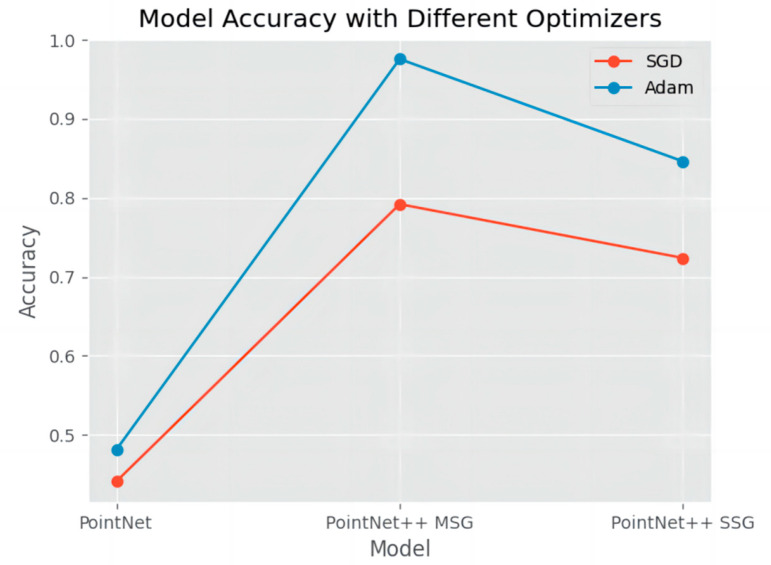
Best Accuracy Achieved by Different Model and Optimizer Combinations.

This experiment primarily adjusted the training model, optimization strategy, and the number of input point cloud points, recording their respective best training accuracy to facilitate the selection of the training model and optimization strategy. The performance of different optimizers (SGD and Adam) under three model architectures (PointNet, PointNet++ MSG, and PointNet++ SSG) was compared. The results are presented in Figure 5.

The results indicate that when using SGD, the best accuracy achieved by the three models was as follows: PointNet: 0.440559, PointNet++ MSG: 0.791958, and PointNet++ SSG: 0.723776, suggesting that SGD’s performance across these models is relatively modest. In contrast, when using the Adam optimizer, model accuracy improved significantly, with PointNet reaching 0.480769, PointNet++ MSG achieving 0.975806, and PointNet++ SSG attaining 0.846154. This demonstrates the advantage of the Adam optimizer in enhancing model accuracy, particularly in the PointNet++ MSG architecture, where it delivered a notable performance boost. These findings suggest that the Adam optimizer provides a more stable and efficient training process for these models, leading to higher classification accuracy. Based on the experimental results, the PointNet model combined with the SGD optimizer performed poorly in tests, while the combination of the PointNet++ MSG method and the Adam optimizer achieved the highest accuracy. Therefore, this study recommends adopting the combination of the PointNet++ MSG model and the Adam optimizer.

Based on the hyperparameter tuning experiments described above, it is concluded that, under the computer configuration and deep learning framework used in this study, the optimal hyperparameter combination should be set as follows: a learning rate of 0.00075, a batch size of 6, and the PointNet++ MSG model selected as the training model.

#### 4.1.4. PointNet++ Road Point Cloud Data Classification Results and Accuracy

The PointNet++ model was trained using the road scene classification dataset. The results show that the average classification category accuracy during the training process was approximately 86.26%. Throughout the training, the highest single-instance accuracy reached about 98.55%, while the best category accuracy was approximately 93.15%. In testing, the model achieved a classification accuracy of 97.41% and a category accuracy of 84.50% on the test set.

**Figure 6:**
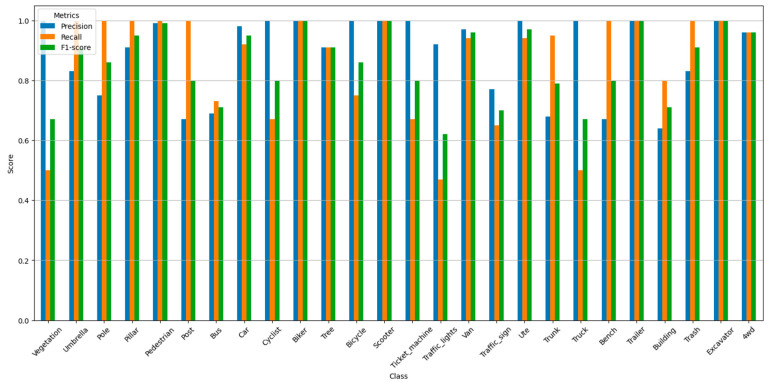
Precision, Recall, and F1 Score by Category.

This study further evaluated the classification accuracy using three metrics: precision (P), recall (R), and F1 score (a harmonic mean of precision and recall). These methods have been detailed in Section 3.5 and will not be reiterated here. For each category, the three metrics were calculated according to the formulas in Section 3.5, and the results are summarized in Figure 6:

This study evaluated the classification performance of different categories, including precision, recall, and comprehensive accuracy. Most categories exhibited high precision and recall, especially categories such as biker, excavator, scooter, and trailer, which showed very high classification accuracy. Some categories, such as building, traffic_lights, and vegetation, however, had lower recall and F1-score, possibly due to the high variability of these categories in the point cloud data or the sparsity of some samples. Overall, the F1-score for most categories was above 0.7, indicating that the model performed relatively well in the classification tasks for most categories. In particular, categories such as car, pedestrian, and van maintained high levels of both precision and recall, demonstrating good classification capabilities.

## 5. Discussion

### 5.1. Generalization Issues Under Cross-Dataset Training

This study integrates three point cloud datasets, namely ModelNet40, ShapeNet, and the Sydney Urban Objects Dataset, with the aim of simulating the complex characteristics of LiDAR data acquired in urban road scenes under controlled conditions. It is unavoidable that point clouds from different sources exhibit a certain degree of domain discrepancy, which mainly arises from substantial differences in sampling mechanisms, point density distributions, noise characteristics, and object completeness. Specifically, synthetic or CAD based point cloud models are typically characterized by regular and complete geometric structures with relatively uniform sampling, whereas real world LiDAR scans collected from road environments often suffer from occlusion, viewpoint limitations, and sensor noise, resulting in sparse and incomplete point representations.

Previous studies and our empirical observations indicate that deep learning models tend to learn discriminative features more effectively from geometrically complete and densely sampled point clouds, which may lead to a reduced representation capability for sparse or partially observed point clouds. Such data distribution induced feature learning bias can negatively affect the generalization performance of models when applied to real world LiDAR data. To mitigate this issue, a FPS based downsampling strategy was applied to CAD based point cloud samples in order to reduce the discrepancy in point density between synthetic and measured point clouds. Experimental results demonstrate that this strategy helps balance the influence of different data sources during training and improves the model’s adaptability to sparse point clouds.

From another perspective, when unified preprocessing and data augmentation strategies are applied, the heterogeneity among multi source point cloud datasets can be regarded as a beneficial factor for approximating the diversity of sampling conditions encountered in real urban road scenes. LiDAR data collected in practical environments often exhibit varying levels of sparsity and incompleteness due to occlusions, object motion, and changing observation conditions. By organizing and modifying point cloud data from different sources in a controlled manner, the diversity of training samples can be enhanced, thereby contributing to improved robustness and generalization capability of the model in complex real world scenarios. In future work, domain adaptation or domain generalization techniques will be further explored to explicitly reduce cross dataset discrepancies and to improve model transferability to unseen real world point cloud data.

### 5.2. Accuracy Analysis of Terrain Point Cloud Recognition

Due to hardware limitations, the dataset used for training in this study is relatively small, and there exists a class imbalance in the point cloud data across different categories, where the sample sizes for various feature point clouds vary significantly. High precision coupled with low recall often indicates class imbalance. If the number of positive class samples in the dataset far exceeds that of negative class samples, even a model that simply predicts all samples as positive can achieve relatively high precision. However, since the model fails to correctly identify negative class samples, recall remains low. Conversely, low precision but high recall occurs when the test set contains a larger proportion of negative classes. In such cases, the model tends to make conservative predictions, favoring negative class predictions to avoid over-predicting positive classes, which may be aimed at reducing the risk of false positives. As a result, recall is relatively high, but precision suffers.

In either scenario, class imbalance significantly impacts model accuracy. This reality causes the model’s predictions to lean toward categories with larger sample sizes, leading to overfitting and, ultimately, some degree of distortion in the prediction results. Although data augmentation methods were employed during data processing in this study, they were insufficient to compensate for the drawback of limited sample size. Moreover, the insufficient training data volume leads to considerable deviations when the model predicts external data, especially when the point cloud density of the prediction target is much higher than that of the training samples. In such cases, predictions tend to favor categories with a higher average point count in the training dataset. For example, the car category samples in the ShapeNet dataset (with an average point count of around 2000) are closer in point count to the building category samples in the road scene classification dataset (with an average point count of around 1500). This similarity causes car category samples from the ShapeNet dataset to be misclassified as building during recognition.

Beyond these data-related factors, misclassifications also exhibit clear geometry-driven confusion patterns across specific object categories. Two representative types of confusion can be identified. The first involves elongated or pole-like objects, such as traffic signs and traffic lights, which share similar slender vertical structures and limited cross-sectional variation. When sampling is sparse or observations are incomplete, these objects are often represented by a small number of vertically aligned points, making it difficult for the model to distinguish fine-grained structural differences based solely on geometric features. The second type of confusion arises among flattened or horizontally extended objects, such as benches and cars. Under partial observation or certain viewpoints, both categories may exhibit low vertical profiles and dominant planar characteristics, causing the learned representations to emphasize overall shape extent and point density rather than subtle structural cues. These patterns indicate that the observed misclassifications are not random but are strongly associated with shared geometric properties and incomplete local observations, highlighting inherent limitations of geometry-based feature learning under sparse and heterogeneous point cloud conditions.

Although misclassification issues exist to some extent, the model still achieves relatively high accuracy after appropriate hyperparameter adjustments. Therefore, these results suggest that when the dataset has a sufficient number of samples and significant inter-sample variability, the PointNet series of models hold promise for achieving high-precision classification of point cloud data, thereby meeting the diverse needs of point cloud data classification across various fields.

### 5.3. Recognition and Classification of Vehicle Point Cloud Targets

This study successfully implemented target recognition functionality for specific road scene features using a trained model. In general road scenes, vehicles are a common type of feature and represent one of the critical recognition targets in autonomous driving technology. Therefore, this study emphasizes the use of vehicle point cloud data recognition accuracy as an evaluation criterion to further assess the overall accuracy of the model.

The specific method is as follows: A total of 500 vehicle point cloud samples that were not involved in training were randomly selected from the ShapeNet and ModelNet40 datasets. Additionally, 150 random category samples were randomly selected from each dataset. The two types of data were shuffled and saved in the same folder to form a target recognition dataset. All data in the dataset were then input into the model in random order for individual recognition, and the model’s judgment category for each sample was recorded. The accuracy of these judgments was analyzed separately. In this way, the vehicle recognition task was approximated as a binary classification task, where the recognition results for each object were categorized as either vehicle or other categories. The evaluation methods, including precision, recall, and F1 score, were also applied for assessment.

**Figure 7:**
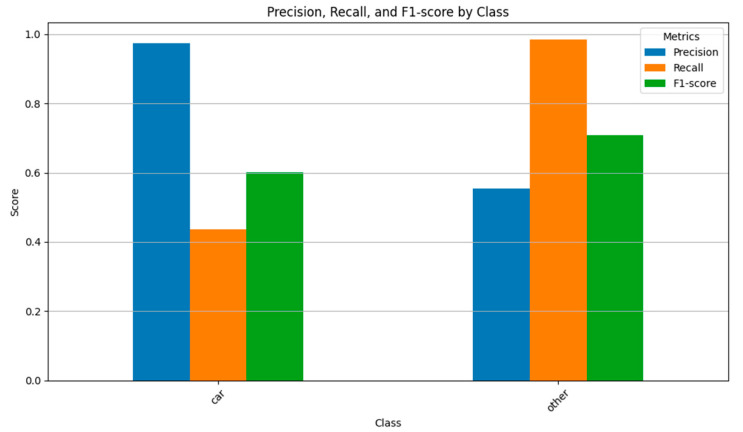
Vehicle Point Cloud Recognition Accuracy.

For the car category, the precision is 0.974490 and the recall is 0.435576. This indicates that the model successfully identified most of the car category samples with minimal misclassification. However, some car category point clouds were misclassified into other categories, resulting in a failure to capture all car category point clouds. The F1 score, which balances precision and recall, is 0.602049. For the other category, the precision is 0.554054 and the recall is 0.984000. This suggests that while a portion of the other category samples were misclassified, a significant proportion were correctly identified. The model successfully captured most of the other category point clouds, with only a small number misclassified into other categories. The F1 score for the other category is 0.708934 (Figure 7).

The results demonstrate that the model is generally capable of recognizing point clouds of the car category, while also revealing certain performance constraints under the current experimental setup. In the present implementation, object-level recognition is achieved at an approximately one-second time scale per instance, which is sufficient for offline analysis and preliminary scene understanding. It is worth noting that this performance is obtained under a relatively modest training environment and limited dataset size. With improved computational resources and optimized deployment platforms, the inference latency is expected to be further reduced to sub-second levels, thereby better satisfying real-time application requirements. In addition, the current study focuses on static object recognition and does not explicitly model temporal continuity. Incorporating temporal information, such as multi-frame point cloud sequences combined with auxiliary data sources (e.g., inertial navigation or tracking algorithms), represents a natural direction for future work to enable object tracking, trajectory estimation, and motion analysis.

## 6. Conclusions

This study systematically integrates the multimodal data advantages from three benchmark datasets—Princeton ModelNet40, ShapeNet, and Sydney Urban Objects Dataset—to establish a comprehensive classification framework for diverse road scene point cloud features. By constructing category-specific point cloud representations of various road elements, we developed a novel point cloud classification dataset that accurately captures the characteristics of real-world road environments. To address critical challenges including occlusion-induced noise and data incompleteness in measured road point clouds, we implemented an advanced data augmentation pipeline: (1) Farthest Point Sampling (FPS) was employed to preserve essential geometric features while mitigating overfitting risks; (2) Rigid transformations incorporating Z-axis rotations, spatial translations, and isotropic scaling were applied to enhance data diversity and improve model generalization; (3) Gaussian noise injection was introduced to realistically simulate environmental disturbances in field measurements, thereby increasing the physical authenticity of training samples. Building upon these foundations, we enhanced the PointNet++ architecture by integrating a point completion mechanism into the data preprocessing module, enabling effective adaptation to the specific requirements of our custom dataset for both model training and prediction tasks.

Subsequently, we implemented the multi-scale grouping (MSG) and single-scale grouping (SSG) classification methodologies within the PointNet++ framework to conduct comprehensive deep learning analysis and predictive modeling on our road scene dataset. The experimental results demonstrated robust performance metrics: (1) The model achieved a mean training classification accuracy of 86.26% (±1.23%), with peak single-instance accuracy reaching 98.54%; (2) Optimal category-specific accuracy attained 93.15% across the training epochs; (3) During testing evaluation, the framework maintained high generalization capability, yielding 97.41% overall classification accuracy with category-level accuracy stabilizing at 84.50%. These quantitative outcomes confirm the successful realization of our primary research objective regarding road scene feature classification in point cloud data, while simultaneously establishing substantive benchmarks for subsequent investigations in point cloud processing and practical applications.

## Figures and Tables

**Table 1:** Organization of point cloud data in the ModelNet40 dataset.

Fields	x	y	z	Nx	Ny	Nz
Description	Three-dimensional spatial coordinates			Three-dimensional spatial normal vector		
Format	float32 ×3			float32 ×3		
Delimiter	The point cloud data in the ModelNet dataset uses the English character “,” as the delimiter					

**Table 2:** Organization of Point Cloud Data in the Sydney Urban Objects Dataset.

Fields	t	Intensity	Id	x, y, z	Azimuth	Range	Pid
Description	Number of microseconds since the epoch	Laser return intensity (0–255)	Laser ID (Velodyne has 64 unique laser IDs)	3D point spatial coordinates	Horizontal azimuth angle, in radians, after correction	Laser return range (meters), corrected	Point IDs from a 360-degree scan
Format	int64	uint8	uint8	float32 x3	float32	float32	int32

**Table 3:** Sources of Sample Data in the Road Scenario Classification Dataset.

Sample Sources	Categories
Sydney Urban Objects Dataset	All Sample Files
ModelNet40	person, bench
ShapeNet	car

**Table 4:** Computer Configuration and Deep Learning Environment Overview.

Category	Parameters and Versions
CPU	lntel(R) Core(TM) i7-10750H (Santa Clara, CA, USA)
GPU	NVIDIA GeForce GTX1650 Ti (Santa Clara, CA, USA)
Memory	16 GB
Operating System	Windows 10
Deep Learning Frameworks	PyTorch 1.12.0

**Table 5:** Quantitative Analysis of Model Advantages and Disadvantages under Different Learning Rates (+: Advantage; ++: Significant advantage; ±: Mediocre or unpredictable; −: Disadvantage; − −: Significant disadvantage).

Learning Rate	0.00025	0.0005	0.00075	0.001	0.00125	0.0015	0.00175
Advantages and Disadvantages	+ High training accuracy + High test accuracy − Low category accuracy − Small learning rate, prone to falling into local optima	++ Highest training accuracy + High test accuracy − Small learning rate, prone to falling into local optima	+ High training accuracy ++ Highest test accuracy + High category accuracy	± Moderate training accuracy ± Moderate test accuracy ++ Highest category accuracy	− Low training accuracy − Low test accuracy − Low category accuracy	− Low training accuracy −− Lowest test accuracy − Low category accuracy − Large learning rate, difficult to converge	− Low training accuracy − Low test accuracy −− Lowest category accuracy − Large learning rate, difficult to converge
Score	0	2	4	2	−3	−5	−5

**Table 6:** Best Accuracy Achieved Under Different Batch Sizes.

Batch	4	6	8
Best Accuracy	0.975806	0.987719	0.973958

## Data Availability

No new data were created or analyzed in this study.
